# Emergency Department Visits for Sexual Assault: A Retrospective Review of Injury Reports

**DOI:** 10.3390/healthcare14101307

**Published:** 2026-05-12

**Authors:** Josep Ramis, Marina Pinedo Terrados, Alberto Pajares Fernández, Joan Masip, Ana Santurtún

**Affiliations:** 1 Juzgado de Primera Instancia e Instrucción núm. 4 de Plasencia, 10600 Cáceres, Spain; joseprgcia@gmail.com; 2 Unidad de Medicina Legal, Departamento de Fisiología y Farmacología, Universidad de Cantabria, 39011 Santander, Spain; marina.pinedo@alumnos.unican.es; 3 Hospital Universitario Marqués de Valdecilla, 39008 Santander, Spain; alberto.pajares@scsalud.es; 4 Hospital Sierrallana, 39300 Torrelavega, Spain; joan.masip@scsalud.es; 5 Marqués de Valdecilla Research Institute (IDIVAL), 39011 Santander, Spain

**Keywords:** sexual assault, emergency department, injury report, substances, chemical submission

## Abstract

**Highlights:**

**What are the main findings?**

- Sexual assaults occur especially during weekends, and the perpetrator is usually someone known to the victim.
- Victims of sexual assault are increasingly younger.
- Victims of sexual assault have very frequently consumed alcohol or other drugs of abuse.
- Women who have consumed alcohol are less likely to report the assault after experiencing sexual violence.
- There is relevant information for judicial proceedings that is not consistently collected during the initial medical evaluation.

**What are the implications of the main findings?**

- Understanding the demographic profile of victims and the temporal pattern of sexual assaults is useful for tailoring interventions.
- Preventing sexual assaults requires knowledge of the characteristics of the assaults, the perpetrators, and the victims.
- The data and samples collected during the initial medical care of a sexual assault victim will shape the entire judicial process.

**Abstract:**

**Objectives**: This study aims to characterize sexual assaults through the analysis of demographic, clinical, and medico-legal variables and to identify areas for improvement both in the care pathway and in the collection of information relevant to judicial proceedings. **Methods**: A retrospective review of injury reports issued by the reference hospital in Cantabria (northern Spain) between 2021 and 2024 was conducted. Variables related to the victim and the assault (including its temporal and spatial characteristics) were extracted, and both descriptive and analytical statistical methods were applied. **Results**: The results showed that 57% of reported sexual assaults occurred in a private residence, and in 68% of cases, the victim had voluntarily met with the aggressor. Temporally, incidents took place predominantly during early morning hours and on weekends, and most victims sought medical care within the first 24 h. Notably, 52% had consumed drugs or alcohol, 22% reported memory gaps suggestive of chemical submission, and victims who had consumed alcohol were less likely to express an intention to press charges (*p* = 0.005). In over half of the reports, the information provided was insufficient to properly interpret the results of biological trace analyses. **Conclusions**: Identifying common variables related to both the victims and the circumstances of sexual assault is essential for developing effective prevention and intervention strategies. Furthermore, the information to be included in injury reports for sexual assault should be standardized at the national level, as shortcomings in data collection during the initial medical assessment (which is highly relevant for judicial processes) highlight the need to raise awareness among emergency care professionals.

## 1. Introduction

Sexual violence constitutes a violation of the physical integrity and autonomy of victims, and it has long-lasting adverse consequences for both physical and mental health [1,2].

When a victim of sexual assault arrives at the hospital, it is essential to ensure comprehensive medical care through the joint and coordinated work of the attending physician and the forensic doctor. The care process must be adapted to the trauma experienced, ensuring professionalism, privacy, and dignity, while at the same time maintaining procedural rigor from a medico-legal perspective in order to optimize evidentiary value in judicial proceedings [3].

This study, based on injury reports from a hospital in northern Spain, seeks to characterize sexual assaults by analyzing variables of demographic, clinical, and medico-legal interest and to understand the victims’ health condition and intentions at the moment closest to the alleged assault. Furthermore, the discussion identifies aspects that require improvement in the care process and in the collection of information relevant to judicial proceedings. It is important to note that visits to the emergency department are often the first (and frequently the only) means used by victims to seek help.

## 2. Materials and Methods

This work was approved by the CEIm of Cantabria (Internal code: 2025.171).

### 2.1. Data Source

The data source consisted of the injury reports for sexual assault issued by the Marqués de Valdecilla University Hospital (HUMV) for the period 2021–2024.

Injury reports are the medico-legal documents used in Spain to notify the judicial authorities of any injury that may constitute a criminal offense. The purpose of this document is to ensure that the authority is informed of the incident and its consequences, enabling the corresponding investigation to be undertaken and, where appropriate, allowing for the adoption of the necessary measures regarding the perpetrator.

In Spain, Article 259 of the Criminal Procedure Act (Ley de Enjuiciamiento Criminal, LECrim) establishes the general obligation for all citizens to report criminal acts. In the case of physicians, they are specifically required to report their provision of care to injured individuals (LECrim, Article 262). For this reason, it is expected that all victims of sexual assault treated at the HUMV would be recorded in the data source used. However, it should be noted that the prosecution of sexual assaults generally requires a complaint by the aggrieved person or their legal representative or a formal accusation by the Public Prosecutor (Criminal Code, Article 191).

### 2.2. Study Variables

The anonymized injury reports were individually reviewed, and general variables related to the victim, as well as temporal and medico-legal aspects, were extracted.

From a demographic perspective, the sex and age of the alleged victim were recorded. To establish the contextual framework, information was collected on the location of the alleged assault, the type of victim–perpetrator relationship, the number of individuals involved, and whether the encounter had been voluntary.

Regarding temporal variables, the following were extracted: the time at which the alleged victim arrived at the hospital; the time when the alleged assault occurred (categorized as: morning [06:00–12:59], afternoon [13:00–20:59], night [21:00–00:59], and overnight [01:00–05:59]); the day of the week; and the month of the year.

Medico-legal variables included the characteristics of the assault (type of violence and the presence or absence of penetration). In addition, the presence of other physical injuries, prior consumption of alcohol or drugs by the alleged victim, suspected drug-facilitated sexual assault, potential loss of evidence, condom use, the date of the last consensual sexual intercourse, and whether or not the victim intended to file a report were analyzed.

### 2.3. Statistical Analysis

A descriptive and analytical analysis was performed. Quantitative variables were described using the mean and median, along with measures of dispersion and percentiles, while qualitative variables were expressed as absolute frequencies and percentages. For inferential analysis, comparisons of qualitative variables were conducted using the chi-square test. Quantitative variables showed a non-parametric distribution, as confirmed by the Shapiro–Wilk test (*p* < 0.001); therefore, the Mann–Whitney U test was used to compare two independent groups, while the Kruskal–Wallis test was applied when comparing three or more groups. Jamovi 2.6.44 [4], a free and open-source software package, was used as the statistical analysis tool.

## 3. Results

Between 1 January 2021 and 31 December 2024, a total of 106 sexual assault reports were issued at the HUMV, 102 involving women and 4 involving men. The mean age of the alleged victims was 23.6 years (SD: 12.4), the median age was 20.5 years (P10: 12.5–P90: 43), the youngest individual was a 2-year-old girl, and the oldest was a 63-year-old woman; Table 1 shows some of the main results, stratified by age group.

All reports included the victim’s account of the events. In some cases, the physician documented the victim’s exact words in quotation marks regarding the type of violence or the chronology of the events.

The location of the alleged assault was specified in 87.7% of the reports. Most assaults occurred inside a dwelling (57% of those in which the information was available). When grouped together with other building spaces (entrance halls, stairways, garages), the percentage increased to 61.3%. The second most frequent locations were public spaces (streets, parks, beaches), accounting for 14.0% of alleged assaults. Bars and nightclubs were the third most common setting (8.6%), followed by vehicles (7.5%).

In 67.7% of cases, the victim had voluntarily met with the perpetrator.

The alleged victims did not know the perpetrator in 24.3% of the cases. In 13.6% of the cases, the perpetrator was a partner or ex-partner of the victim; in 8.7%, a family member; in 8.7%, a friend; and in 8.7%, someone they had previously met online. The remaining perpetrators were acquaintances without a close relationship, a category that included co-workers, individuals from the victim’s social circle, neighbors, and others.

Regarding the number of participants, 88.8% of cases involved a single perpetrator; 6.1% involved two; 2.0% involved three; 1.0% involved six; and in 2.0% of cases, multiple unspecified perpetrators were reported.

### 3.1. Temporal Analysis

**Figure 1:**
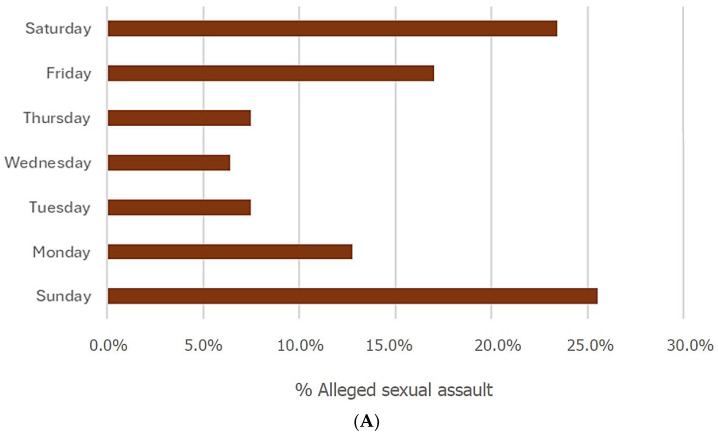

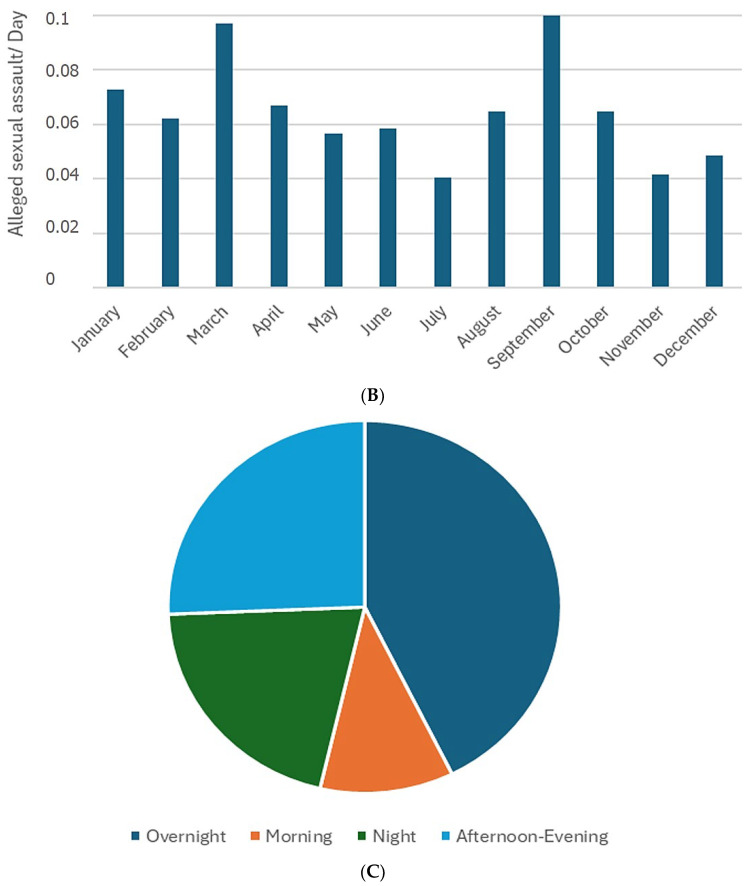
Temporal pattern of emergency department visits for sexual assault. Note: (**A**)—Percentage of alleged assaults by day of the week. (**B**)—Average number of alleged assaults per day for each month of the year. (**C**)—Percentage of alleged sexual assaults according to the time of day.

The date of the alleged sexual assault was recorded in 88.7% of the reports. Of these, 48.5% of the alleged assaults occurred during the weekend (Saturday and Sunday), and 66.0% took place between Friday and Sunday (Figure 1A). The difference by day of the week was statistically significant (χ^2^ = 24.7; *p* < 0.001).

The highest daily number of sexual assault reports was issued in March and September (Figure 1B).

Regarding the time of the alleged assault, 11.0% of victims stated it occurred in the morning, 20.7% in the afternoon, 25.6% at night, and 42.7% in the early morning hours (Figure 1C). The difference in distribution across times of day was statistically significant (χ^2^ = 17.3; *p* < 0.001). However, no statistically significant association was found between time of day and the age of the alleged victims (*p* = 0.123).

Regarding the time elapsed between the assault and the medical consultation, 12.1% of visits were made within two hours or less of the alleged assault; 68.7% occurred more than two hours but within one day; 15.2% took place between one and six days afterwards; 2% took place between one week and one month afterwards; and 2% took place more than one month after the alleged assault.

### 3.2. Medico-Legal Findings

The type of sexual violence was recorded in 83.0% of the reports, classifying it as vaginal, anal, oral, or manual; some patients reported having suffered more than one type of violence.

Among these, 75% of patients described vaginal violence, 27.3% anal, 19.3% oral, and 19.3% manual. Of these cases, 35.2% involved two or more types of violence. Conversely, in the 17.0% of reports in which this variable could not be recorded, 77.0% of the alleged victims stated that they did not remember the type of assault.

In 46.2% of the sexual assault reports, the presence of gynecological injuries was documented (either described in the report or identified in accompanying diagrams), and 59.4% indicated that penetration had occurred.

Regarding the possible loss of evidence, two variables were considered: a dichotomous one indicating whether or not evidence loss had occurred and a second one assessing what actions the patient had undertaken after the alleged assault and before arriving at the hospital. The first variable was recorded in 75.5% of reports, and among these, 81.3% of alleged victims stated that evidence may indeed have been lost.

Information on the type of action performed by the victim was included in 61.3% of injury reports. Among these, 43.1% of alleged victims specified that they had showered; 80% reported having urinated; 58.5% had changed clothes (5.3% of whom provided the garments); and 1.5% reported brushing their teeth. In 7.7% of cases, the possible loss of evidence was due to several weeks having passed since the events.

Information about the last consensual sexual intercourse was not included in 51.9% of reports. Among the 49 patients for whom this variable was available, 17 had never engaged in sexual intercourse prior to the alleged assault.

Among cases describing vaginal, anal, or oral penetration in which the victim retained memory of the assault, 7.1% of reports indicated that the perpetrator used a condom, and 51.8% stated that no condom was used, while this variable was missing in 41.1% of reports.

In 36.8% of reports, it was specified that, in addition to the sexual assault, the victim presented with physical injuries. Additionally, one case involved self-inflicted injuries following the assault, and another involved a patient who experienced an anxiety attack.

A total of 49.1% of alleged victims reported alcohol consumption, 14.2% reported use of other drugs of abuse, and 11.3% reported consumption of both.

In 93.4% of reports, it was noted whether or not there was suspicion of drug-facilitated submission. Among these, such suspicion existed in 22.2% of cases.

Information about the victim’s intention to press charges was included in 84.9% of reports; among these, 82.2% stated their intention to file a complaint. While age did not influence the intention to press charges (*p* = 0.605), it is noteworthy that victims who had consumed alcohol were less likely to intend to press charges (χ^2^ = 7.83; *p* = 0.005).

## 4. Discussion

Most sexual assault reports involve young women; in this study, the median age was 20.5 years. A 2025 study analyzing the age at which individuals experienced their first episode of sexual violence across numerous countries found that it usually occurred during childhood or adolescence [5]. In some countries, victims have been reported to be increasingly younger [6], and this is also observed in Spain when comparing our results with earlier studies. Although there are few studies conducted in Spain on sexual violence based on medical data, a study at a hospital in Barcelona reported that women who attended between 2005 and 2008 had a median age of 25 years [7].

Several studies have examined risk factors for perpetration of sexual violence among adolescent males, identifying alcohol consumption, previous sexual violence or intentions to commit it, exposure to physical or sexual child abuse, delinquent behavior, exposure to parental violence or family conflict, sexual risk-taking, misperception of sexual cues, and exposure to sexually explicit media (pornography) [8]. Risk factors for sexual victimization among adolescent girls have been linked to having multiple partners in short periods of time, engaging in sex with strangers, not using condoms, being intoxicated during sexual encounters, and having experienced prior abuse. The authors emphasize that identifying risk factors in victims does not imply attributing responsibility but rather aims to provide empirical data to guide prevention and appropriate intervention [9].

In addition, the increase in demand for healthcare services at younger ages may be due to greater awareness of available support resources after sexual assault and increased public consciousness regarding sexual violence [10].

In relation to this, we consider it important to highlight that during the four-year study period, four male victims were attended to (two adults), which leads to the question of whether there was only one male victim per year. Sexual violence against men remains a taboo subject, leading to under-detection and under-reporting. In this respect, the role of healthcare centers is fundamental. Hospitals need to develop injury report forms specifically adapted for male victims (currently nonexistent) and establish intervention protocols tailored to this population, as the lack of such resources may heighten feelings of fear and shame when seeking help or reporting the assault.

A majority of those who reported having suffered sexual assault stated that the encounter had initially been voluntary and that they knew the perpetrator (or even had a close relationship). This is consistent with studies conducted in the United States [11] and in European countries [12,13]. Jänisch et al. [13], compiling information from the reports of the Institute of Forensic Medicine in Hanover over a three-year period, found that only 12.6% of perpetrators were strangers to the victims, and in 24.3%, they were family members, partners, or ex-partners.

The temporal pattern of sexual assaults has seldom been analyzed in the literature. In our results, no seasonal pattern was detected, but there was a clear predominance of assaults during the early morning hours and on weekends.

The temporal concentration of assaults may be linked to another variable studied: substance use, mainly alcohol, which appeared in the majority of injury reports. Victims who had consumed alcohol were less likely to express an intention to press charges, which is probably related to uncertainty about the events (memory impairment) and social stigma. For professionals providing initial care and support, it is important to emphasize that intoxication (depending on its level) may raise questions about the presence or validity of consent for sexual activity.

Suspicion of drug-facilitated sexual assault was present in 22% of cases. The literature shows that percentages vary by geographical region and study period [13,14,15]. Saint-Martin et al. [15] conducted a study in Tours, France (1996–2002), where suspicion of drug-facilitated submission was noted in only 2.9% of cases. However, recent studies in New York [11] and Dublin [12] report figures close to those found in our study (22.9% and 20.2%, respectively).

In Spain, a study focusing on toxicological analysis in suspected drug-facilitated sexual assault concluded that the most frequently detected substance was alcohol (52.9%), followed by other drugs of abuse (37.2%) [16].

From a medico-legal perspective, delays in reporting and seeking medical care in sexual offenses pose serious challenges that can hinder the prosecution of perpetrators and prevent victims from receiving necessary support [17]. In our study, although data are limited to those who chose to seek medical assistance, more than 80% did so within the first 24 h. This may be considered a positive finding, as various authors have reported much longer delays, often exceeding one week [18,19].

This percentage may also be related to the fact that more than one-third of individuals reporting sexual assault presented with injuries from other forms of physical violence.

At this point, we consider it essential to stress the need to train and sensitize healthcare professionals responsible for assisting victims about the importance of documenting the victim’s account and conducting a guided anamnesis. Information such as the time since the last consensual sexual intercourse or the presence or absence of condom use by the perpetrator is missing in many reports, yet these details are fundamental for interpreting forensic genetic laboratory results. In addition, consistency in the account within judicial proceedings is crucial for evaluating testimonial credibility, and the hospital is often where the victim narrates the events for the first time.

Finally, this study has some limitations that should be addressed in future research. First, the retrospective design means that some variables of interest could not be analyzed because they were not systematically recorded by physicians, and in some cases, injury reports were incomplete. Second, the study was conducted in a single-center setting, which limits the sample size. Third, the information collected is limited to data from victims who presented to the hospital; it is well known that many individuals still do not seek medical assistance after experiencing sexual assault.

## 5. Conclusions

The main findings of this study are as follows: (1) In most assaults, the victim’s encounter with the perpetrator was initially voluntary. (2) From a temporal perspective, sexual assaults occur predominantly during the early morning hours and on weekends, and healthcare is sought within a short period of time (less than one day). (3) Substance use among the alleged victims is very common, and 22% present amnesic gaps with suspicion of drug-facilitated submission. (4) More than one-third of individuals who reported having suffered a sexual assault also presented with physical injuries. (5) The data collection process in injury reports should be standardized. Despite the medico-legal importance of obtaining information about the last consensual sexual intercourse, this information was missing in 52% of sexual assault reports, and in 41% of cases involving vaginal, anal, or oral penetration, the use of a condom was not documented.

Regarding this last point, it should be noted that in hospitals in Cantabria, the information included in injury reports for sexual assault varies, and it also differs among hospitals in other regions of Spain. Considering the information that is relevant to judicial proceedings, the injury report should be standardized at the national level and accompanied by guidelines so that attending physicians record all information of interest for the judicial process.

It is known that many individuals who experience a sexual assault seek care at the hospital, reporting other somatic or mental health concerns. Given the difficulty of recognizing and disclosing having been a victim of sexual assault (particularly considering that in many encounters the victim initially met with the perpetrator voluntarily), some of the variables identified in this study may help clinicians recognize patterns that warrant asking directly about the possibility of sexual assault: a young woman arriving at the emergency department during the early morning hours, who has consumed alcohol, is emotionally distressed, and may present with physical injuries. In this regard, training emergency physicians in risk detection should be a priority.

Understanding the common patterns related to the victims and the characteristics of these types of assault are essential for prevention and intervention strategies. Furthermore, this study identifies shortcomings in data collection during the initial medical assessment that are relevant to judicial proceedings, an aspect that emergency healthcare professionals should be made aware of.

## Figures and Tables

**Table 1:** Distribution of variables by age group among victims of sexual assault.

	Agre Group		
	<25 Years	25–34 Years	>34 Years
Number of patients (n)	70	14	22
Alcohol consumption (%)	45.7%	57.1%	54.5%
Suspected drug-facilitated assault (%)	20.0%	21.4%	22.7%
Assault at night or early morning (%)	47.1%	85.7%	50.0%
Intention to press charges (%)	65.7%	85.7%	72.7%

## Data Availability

Requests to access the datasets should be directed to the corresponding author (ana.santurtun@unican.es).

## Abbreviations

The following abbreviations are used in this manuscript:

CEIm	Comité de Ética de Investigación con Medicamentos
HUMV	Hospital Universitario Marqués de Valdecilla
SD	Standard deviation
P10–90	Percentile 10–90
